# Delirium Tremens Treated by Ammonia

**Published:** 1843-07

**Authors:** 


*Delirium Tremens Treated by Ammonia.*—Dr. Scharn, of Katscher, considers the affection in the light of intoxication at its maximum of intensity, and acting on the faith that ammonia is the most suitable remedy against the effects of ardent spirits, he has recourse to the succinate of that base on the access of the delirium; and he states that he has witnessed the most aggravated cases of the latter succumb to this remedy in a few days.—*Casper's Wochenschrift.*—*London Lancet.*

